# Examination of DNA Methylation Patterns in Children Born Premature with Prenatal Tobacco Smoke Exposure

**DOI:** 10.3390/toxics13090789

**Published:** 2025-09-17

**Authors:** Olivia E. Gittens, Alonzo T. Folger, Xue Zhang, Lili Ding, Nehal A. Parikh, E. Melinda Mahabee-Gittens

**Affiliations:** 1 Medical Scientist Training Program, University of Cincinnati College of Medicine, Cincinnati, OH 45229, USA; gittenoe@mail.uc.edu; 2 Division of Biostatistics and Epidemiology, Cincinnati Children’s Hospital Medical Center, Department of Pediatrics, University of Cincinnati College of Medicine, Cincinnati, OH 45229, USA; alonzo.folger@cchmc.org (A.T.F.); lili.ding@cchmc.org (L.D.); 3 Division of Human Genetics, Cincinnati Children’s Hospital Medical Center, Cincinnati, OH 45229, USA; xue.zhang@cchmc.org; 4 Neurodevelopmental Disorders Prevention Center, Perinatal Institute, Cincinnati Children’s Hospital Medical Center, Department of Pediatrics, University of Cincinnati College of Medicine, Cincinnati, OH 45229, USA; nehal.parikh@cchmc.org; 5 Division of Emergency Medicine, Cincinnati Children’s Hospital Medical Center, Department of Pediatrics, University of Cincinnati College of Medicine, Cincinnati, OH 45229, USA

**Keywords:** DNA methylation, Tobacco Smoking, Preterm Birth, CpG Islands, Reduced Representation Bisulfite Sequencing

## Abstract

Prenatal tobacco smoke exposure (TSE) has been associated with significant alterations in DNA methylation (DNAm), an epigenetic mechanism with potential functional consequences to child development. This pilot study aimed to investigate differential DNAm patterns in preterm children with and without prenatal TSE using reduced representation bisulfite sequencing (RRBS) to interrogate a wider array of sites than in more common approaches, namely microarrays. Buccal swabs were collected from 16 two-year-old children (7 with TSE, 9 without), and DNAm was quantified at over 1.3 million CpG sites. To identify differential DNAm, univariable analyses were first performed and followed by Bayesian beta-binomial hierarchical regression models for sequence count data including adjustment for potential confounders. False Discovery Rate correction was used to account for multiple comparisons. Significant differential methylation was observed at CpG sites within intronic regions of the *CALN1* and *LINGO1* genes and the distal intergenic region of the *TBL1XR1* gene. These findings suggest that prenatal TSE may influence epigenetic regulation in genes involved in neurodevelopment. This study demonstrates the importance of RRBS in identifying novel DNAm changes associated with prenatal TSE and highlights the need for larger studies to validate and expand upon these preliminary findings.

## 1. Introduction

Prenatal tobacco smoke exposure (TSE) is associated with many adverse consequences in childhood and adulthood which include abnormalities affecting neurodevelopment, immune function, and the respiratory and cardiovascular systems [1,2,3,4,5]. The epigenome, which evidence suggests is sensitive to early exposures, comprises chemical signatures on DNA that influence the regulation of gene expression. These epigenetic modifications may carry potentially functional consequences [6]. It is hypothesized that DNA methylation (DNAm), an epigenetic mechanism that is represented by the addition of methyl groups to the cytosine-phosphate-guanine (CpG) sites of DNA, represents an underlying mechanism whereby TSE may exert some of these adverse effects [5,7,8,9,10].

Prior studies indicate that prenatal TSE is associated with several differentially methylated CpGs in newborns [10,11,12,13,14,15,16,17,18]. These studies have also identified CpGs that are shared between children with in utero TSE and adults who were active smokers [19]. Some of the differentially methylated CpGs observed exclusively in newborns were enriched in pathways related to xenobiotic metabolism, suggesting that these TSE-induced DNAm patterns may underlie some of the adverse health effects of TSE on newborns [10,19].

Previous DNAm studies have used techniques such as epigenome-wide DNA methylation analyses (EWAS), whole genome bisulfite sequencing (WGBS), or microarrays such as the Illumina 450 K array or the MethylationEPIC array [8,11,12,13,16,17]. To our knowledge, no studies have used the reduced representative bisulfite sequencing (RRBS) technique, a cost-effective alternative to WGBS [20,21,22], to examine DNAm patterns in children who were born preterm and were exposed to tobacco smoke prenatally. Aside from lower costs, there are other reasons for using RRBS to analyze clinical samples. RRBS requires minimal DNA [23] which may be particularly useful when collecting samples from young children. Compared to microarrays, RRBS is a flexible technique that allows the interrogation of more loci and the potential discovery of novel biomarkers [24,25]. Additionally, RRBS provides interrogation of DNAm patterns with resolution at the single-nucleotide level at gene promotors and CpG islands (CGIs), commonly unmethylated regions of the genome with increased cytosine and guanine content [23,26,27]. As many as 72% of annotated gene promoters are associated with CGIs [28]. CGIs comprise transcription start sites [29] of approximately 60% of coding genes in humans [30]. Thus, CGIs are important genomic regions that warrant evaluation in medical research [23]. To further examine the use of RRBS, we conducted a pilot study in which RRBS was used to examine the associations of prenatal TSE with DNAm patterns. We hypothesized that we would observe differential DNAm patterns in children who had prenatal TSE compared to children who did not have prenatal TSE. We addressed this hypothesis in a pilot study of the Cincinnati Infant Neurodevelopment Early Prediction Study (CINEPS) birth cohort, comparing seven children born preterm who had prenatal TSE to nine children born preterm who did not have prenatal TSE.

## 2. Methods

### 2.1. Study Participants

This study included data from a subsample (n = 16) of child participants from CINEPS, a parent study that prospectively enrolled and collected data and samples from 395 very preterm infants born at ≤32 weeks gestational age (GA) from September 2016 to November 2019. Enrollment occurred at five level III/IV academic or community Ohio-area newborn intensive care units in Cincinnati and Dayton, Ohio, USA. Infants were excluded if they had known chromosomal or congenital anomalies that affected the central nervous system, cyanotic heart disease, or if they were hospitalized and received mechanical ventilation with greater than 50% supplemental oxygen at 45 weeks postmenstrual age. The Cincinnati Children’s Hospital Institutional Review Board approved the study. The other hospitals’ review boards approved the study based on an established reliance agreement. Written informed consent was provided by the caregivers of all infants.

We selected a convenience sample of children who had buccal swabs collected at two years corrected age (n = 35). Of those, we selected children who had maternal self-reports of tobacco use during pregnancy (n = 7 with prenatal TSE, n = 59 with no prenatal TSE). The selected children were then matched based on child demographics (sex, race, ethnicity), and gestational age (22–28 weeks or 29–32 weeks). The seven children with prenatal TSE were matched on sex and gestational age at birth with children who did not have prenatal TSE (n = 9). Thus, 16 total children were included in the analysis.

### 2.2. Buccal Swab Collection

Buccal swabs are well-tolerated and easy to collect in children [31,32]. Further, research has highlighted the unique opportunities for epidemiologic research using DNAm derived from saliva and more specifically buccal samples [32,33,34]. Moreover, two systematic reviews indicate that saliva and other oral samples can be successfully used to identify DNAm markers [35,36]. Using a standardized collection protocol at all study sites, we collected buccal swabs samples from participants using ORAcollect•Dx swab kits containing preservative (ORD-100, DNA Genotek, Ottawa, ON, Canada) [37,38]. Briefly, the buccal swab was placed as far back in the mouth as possible and rubbed back and forth across both sides of the child’s lower gums 10 times. DNA from buccal swabs was extracted using a Promega Maxwell 16 nucleic acid extractor (Promega Corporation, Madison, WI, USA) using the Buccal Swab Purification LEV DNA kit. DNA extraction was performed via magnetic bead-based protocol. Manufacturer’s protocols were followed as written. Following extraction, genomic DNA was rotated overnight to ensure uniform solution and then assayed for concentration, A260/280 ratio, and other QC values on a Lunatic nucleic acid quantification System (Unchained Labs, Pleasanton, CA, USA) [39].

### 2.3. Maternal and Child Assessments

Enrolled mothers provided information on their child’s demographics (i.e., race, ethnicity), mothers’ age, household income, education, and employment status. Each child’s gestational age, birth weight, and Apgar scores were obtained from their electronic medical record. Mothers were asked if they smoked combustible cigarettes during their pregnancy (yes/no) and children whose mothers reported “yes” were considered to have had prenatal TSE.

### 2.4. DNA Core Protocol

RRBS libraries were prepared with the Zymo-Seq RRBS Library Kit (Irvine, CA, USA) using the suggested protocol [40]. Sequencing was performed on the Illumina NovaSeq 6000 platform (San Diego, CA, USA), generating paired-end 100 base reads. Libraries were denatured and loaded onto the flow cell according to the manufacturer’s protocol. A total of 75 million non-directional paired-end reads per sample were generated, ensuring sufficient coverage for methylation analysis.

### 2.5. RRBS Processing

The methodology included quality control procedures and filtering criteria related to coverage. The total number of CpG sites interrogated was 1,340,955. The preparation of RRBS data involved several key steps to ensure high-quality and reliable results [41]. First, the raw sequencing data underwent quality control using FastQC, version 0.11.2 (Cambridge, UK). Second, the data were trimmed using Trim Galore, version 0.6.6 Ref (Cambridge, UK) to remove low-quality bases and adapter sequences, with the trimmed data being reassessed for improved quality. Third, the trimmed reads were then aligned to the reference genome using Bismark, version 0.22.3 (Cambridge, UK) and bowtie 2, version 2.3.3 (Baltimore, MD, USA), generating Binary Alignment Map (BAM) files and alignment reports for each sample. Post-alignment, the BAM files were sorted with samtools 1.9.0 (Cambridge, UK). Coverage thresholds were set; bases that had coverage below 10X and bases that had more than the 99.9th percentile of coverage in each sample were discarded. Sample correlation was examined using clustering; no outlier samples were observed, therefore all samples were included for analysis.

### 2.6. Statistical Analysis

We examined the association between prenatal TSE and DNAm intensity at each CpG site. Univariable analyses were performed because this was a matched cohort study. These analyses were followed by multivariable models to improve the precision of model estimates, conditioning on the potential confounding factors including race, precise gestational age, and child sex. The epigenome-wide analyses were conducted using the R package (version R4.1.1) Dispersion Shrinkage for Sequencing data (DSS, version 2.42.0) [42,43,44,45,46]. The DSS package employs a Bayesian beta-binomial hierarchical model to analyze count-based data from bisulfite sequencing. This method addresses both technical (i.e., sequencing depth) and biological sources of variation as well as the small-sample analyzed; this occurs by accessing a shrinkage procedure that borrows information from CpG sites across the genome to stabilize the estimation of the dispersion parameters and a Wald test procedure to account for the coverage depth and within-group variance. The False Discovery Rate (FDR) *p*-value correction was used to address multiple comparisons. Overall, the analysis interrogated a total of 1,340,955 CpG sites, providing a comprehensive assessment of DNAm patterns associated with prenatal TSE.

The analysis workflow focused on identifying differentially methylated cytosines (DMCs). For each of the CpG sites, the difference in DNAm was calculated as the difference in group mean methylation, interpreted as percent methylation. Annotation of DMCs was performed using CHIPseeker packages, providing insights into the genomic context of the methylation changes. We also used the UCSC genome browser (hg38) to map genomic features associated with significant CpG sites [47].

## 3. Results

### 3.1. Participant Characteristics

Participant characteristics are shown in Table 1. Of the participants with prenatal TSE, 3 (43%) and 4 (57%) were born between 22–28 weeks and 29–32 weeks, respectively. The prenatal TSE and no prenatal TSE groups were similar in the key demographic and clinical characteristics, except for employment status (Table 1).

### 3.2. Differentially Methylated CpG Sites

In the univariable model, prenatal TSE was significantly associated with DNAm intensity at 4 CpG sites (Table 2). Three of these four sites (75%) had lower DNAm levels associated with TSE. After adjusting for the possible confounding factors of child sex, race/ethnicity, and gestational age, only two sites remained statistically significant at the *p* < 0.01 level: CpG positions 72098981 and 77604573 (lowest FDR *p*-value = 0.0002).

## 4. Discussion

DNAm is a major epigenetic mechanism involved in gene regulation [48]. In this pilot study, we used RRBS to identify differentially methylated CpGs in preterm children with prenatal TSE compared to preterm children without prenatal TSE. Compared to WGBS, RRBS is more cost effective and has the potential to uncover novel biomarkers that have not been previously reported [20,21,22]. The latter may explain why the present analysis revealed four CpG sites that were differentially methylated in children with prenatal TSE, that to our knowledge, have not been previously reported as being associated with prenatal TSE. We identified hypomethylation in the range of 38–43% in the intron regions of calneuron 1 (*CALN1*) [49] at CpG positions 72098981 and 72099057 and in the distal intergenic region of transducin β-like 1X-linked receptor 1 (*TBL1XR1*) [50] at CpG position 177000000 and hypermethylation of 29% in the intron region of leucine-rich repeat- and IG Domain-Containing NOGO receptor-interacting protein 1 (*LINGO1*) [51] at the CpG position 77604573.

Concerning the hypomethylation observed in *CALN1,* this gene encodes a protein that is similar to proteins that bind calcium in the calmodulin family [52]. Calcium signaling is important in cellular processes and may be related to cancer progression [53,54,55]. Hypomethylation of *CALN1* has been reported to be associated with bladder cancer in adults [55] and hydroxymethylation of *CALN1* may be a biomarker that is useful for cervical cancer diagnosis and prognosis [56]. Additionally, *CALN1* is found in regions of the brain such as the hippocampus, cortex, cerebellum, and striatum and it may play a role in memory, learning, and the physiology of neurons [49,52]. Finally, and of particular significance to this pilot study, *CALN1* has been found to be associated with the average daily number of cigarettes smoked among cohorts with chronic obstructive pulmonary disease [57].

We also observed hypomethylation of *TBL1XR1*. The protein encoded by *TBL1XR1* localizes to the nucleus in most tissues, interacts with histones H2B and H4, and is involved in transcriptional activation [50,58]. Mutations in and abnormal expressions of *TBL1XR1* have been associated with autism spectrum disorders, intellectual disability [59] and some cancers [60]. A heterozygous missense mutation in *TBL1XR1* has also been found to cause Pierpont Syndrome, a condition associated with distinctive craniofacial features, global developmental delay [61], epilepsy, and neurodevelopmental disorders including attention-deficit hyperactivity disorder [62].

We observed hypermethylation of *LINGO1*. This gene encodes a transmembrane protein that is expressed in oligodendrocytes and neuronal cells found in the central nervous system [51]. Specifically, high expression of *LINGO1* has been found in the hippocampus, neocortex, and thalamus and lower levels of expressions have been found in the spinal cord and cerebellum [63,64]. *LINGO1* plays a role in the regulation of CNS processes including myelination, oligodendrocyte differentiation, axon regeneration, and neuronal survival [51]. Mutations of *LINGO1* are associated with intellectual disability, microcephaly, speech and motor delay [65].

Although research on the CINEPS cohort has not evaluated if DNAm patterns are associated with neurodevelopmental outcomes, examination of the full CINEPS cohort indicates that prenatal TSE is directly associated with diffuse white matter abnormality, decreased brain tissue volumes, and high global brain abnormality scores [66]. These sensitive measures indicate that prenatal TSE is associated with abnormal brain development and injury in infants. Further, another research study on the CINEPS cohort found that prenatal TSE is associated with lower cognitive scores at 2-years corrected age [67]. Thus, future work should evaluate if specific epigenetic mechanisms and related responses to the environment are associated with neurodevelopmental impairments or other long-term outcomes. Additionally, our study’s findings should be analyzed in future research to assess the reproducibility and clinical findings associated with these results.

### Limitations

While this exploratory pilot study is unique in that we used RRBS to assess epigenetic changes due to prenatal TSE in children born preterm, this study had several limitations. The sample size was small and thus not powered to detect small effects. Nevertheless, even with this small sample size, we identified CpG sites through RRBS that differ from prior work with standard microarray chips. In addition, our sample was matched on factors that could confound the observed associations (e.g., race, gestational age). Thus, the study methods using RRBS and our results provide an important formative basis for future, larger studies to examine our findings. These studies should consider the use of RRBS to compare DNAm patterns in large cohorts of children with and without TSE that are carefully matched on sociodemographic characteristics and birth histories. RRBS should be considered in the future as it is a cost- and time-effective sequencing method that has been used to successfully identify and provide preliminary insights into DNAm patterns in smaller pilot studies [68,69,70]. In this study, prenatal TSE was self-reported by mothers and was not biochemically confirmed with cotinine, a nicotine metabolite [71]. However, prior work indicates that there is fairly high concordance between maternal reports of tobacco use during pregnancy and cotinine measures [72,73,74]. Moreover, our prior research on the CINEPS cohort confirms that prenatal TSE reports by mothers are strongly associated with objective measures of neonatal brain injury, abnormal development, and cognitive delay in children [66]. This prior work underscores our confidence in the self-reports of prenatal tobacco use by CINEP mothers that were used to assess prenatal TSE among children in this pilot study. Nevertheless, to evaluate the role that prenatal TSE has on children, future work should biochemically verify prenatal TSE with cotinine and collect data on the timing, amount, and type of tobacco product used by mothers and household members during the prenatal period. Finally, the use of the CINEPS cohort limits the generalization of these findings to children who were not born preterm or who were born in other settings.

## 5. Conclusions

In this novel pilot study of children born preterm, we used RRBS to analyze DNAm patterns associated with prenatal TSE. Using RRBS, we identified four CpG sites that showed differential methylation patterns which have not been previously reported. Given the advantages of using RRBS and the wide range of conditions associated with the CpG sites we found, these findings should be further investigated in large prospective cohorts to add to the literature about potential mechanisms and consequences of prenatal TSE.

## Figures and Tables

**Table 1:** Characteristics of the study sample.

Child Characteristics	Prenatal TSE *n* = 7	No Prenatal TSE *n* = 9
Child Sex—Female	5 (71%)	7 (78%)
Child Race/Ethnicity White, non-Hispanic Black, non-Hispanic Mixed White and Black, non-Hispanic Unknown race	2 (28.5%) 2 (29%) 1 (14%) 2 (29%)	3 (33%) 5 (56%) 1(11%) 0 (0%)
Gestational Age, weeks 22–28 weeks 29–32 weeks	3 (43%) 4 (57%)	4 (44%) 5 (56%)
Birth weight Z-score, median (range)	−0.30 (−1.47, 0.69)	−0.49 (−1.8, 0.95)
Small for gestational age	1 (14.4%)	2 (22.2%)
Apgar score < 5 at 5 min	2 (33.3%)	1 (11.1%)
Maternal Characteristics		
Maternal age >21 years old	7 (100%)	9 (100%)
Household income * >$100,000 $40,000–$99,999 <$40,000	1 (16.7%) 1 (16.7%) 4 (66.7%)	1 (12.5%) 1 (12.5%) 6 (75.0%)
Education ** Tertiary education High school diploma or GED Less than HS or less than GED	4 (80.0%) 1 (20.0%) 0	7 (77.8%) 0 2 (22.2%)
Employment status Full-time Part-time Student Stay at home caregiver Unemployed/receives a pension	0 0 0 2 (40.0%) 3 (60.0%)	3 (33.3%) 0 2 (22.2%) 2 (22.2%) 3 (33.3%)

* Data missing for one participant with prenatal TSE and one with no prenatal TSE. ** Data missing for two participants with prenatal TSE.

**Table 2:** Associations of prenatal TSE with two-year corrected age DNA methylation when compared to no prenatal TSE.

Chromosome	CpG Position	Mapped Gene	Methylation Difference%	Location	*p*-Value	False Discovery Rate
7	72098981	*CALN1*	−38.2	Intron	4.87 × 10^−9^	0.000272
7	72099057	*CALN1*	−42.8	Intron	1.79 × 10^−5^	0.028501
15	77604573	*LINGO1*	29.0	Intron	2.83 × 10^−9^	0.000192
3	177000000	*TBL1XR1*	−41.5	Distal Intergenic	8.11 × 10^−6^	0.019098

## Data Availability

The datasets used and/or analyzed for this study are available from Dr. Parikh on reasonable request. The data are not publicly available due to privacy restrictions.

## References

[1] Chen D., Niu Q., Liu S., Shao W., Huang Y., Xu Y., Li Y., Liu J., Wang X., Yang H. (2023). The correlation between prenatal maternal active smoking and neurodevelopmental disorders in children: A systematic review and meta-analysis. BMC Public Health.

[2] Miyake K., Kushima M., Shinohara R., Horiuchi S., Otawa S., Akiyama Y., Ooka T., Kojima R., Yokomichi H., Yamagata Z. (2023). Maternal smoking status before and during pregnancy and bronchial asthma at 3 years of age: A prospective cohort study. Sci. Rep..

[3] Raghuveer G., White D.A., Hayman L.L., Woo J.G., Villafane J., Celermajer D., Ward K.D., de Ferranti S.D., Zachariah J., American Heart Association Committee on Atherosclerosis, Hypertension, and Obesity in the Young of the Council (2016). Cardiovascular Consequences of Childhood Secondhand Tobacco Smoke Exposure: Prevailing Evidence, Burden, and Racial and Socioeconomic Disparities: A Scientific Statement From the American Heart Association. Circulation.

[4] McEvoy C.T., Spindel E.R. (2017). Pulmonary Effects of Maternal Smoking on the Fetus and Child: Effects on Lung Development, Respiratory Morbidities, and Life Long Lung Health. Paediatr. Respir. Rev..

[5] Braun M., Klingelhofer D., Oremek G.M., Quarcoo D., Groneberg D.A. (2020). Influence of Second-Hand Smoke and Prenatal Tobacco Smoke Exposure on Biomarkers, Genetics and Physiological Processes in Children-An Overview in Research Insights of the Last Few Years. Int. J. Environ. Res. Public Health.

[6] Jones D.E., Park J.S., Gamby K., Bigelow T.M., Mersha T.B., Folger A.T. (2021). Mental health epigenetics: A primer with implications for counselors. Prof. Couns..

[7] den Dekker H.T., Burrows K., Felix J.F., Salas L.A., Nedeljkovic I., Yao J., Rifas-Shiman S.L., Ruiz-Arenas C., Amin N., Bustamante M. (2019). Newborn DNA-methylation, childhood lung function, and the risks of asthma and COPD across the life course. Eur. Respir. J..

[8] Cosin-Tomas M., Cilleros-Portet A., Aguilar-Lacasana S., Fernandez-Jimenez N., Bustamante M. (2022). Prenatal Maternal Smoke, DNA Methylation, and Multi-omics of Tissues and Child Health. Curr. Environ. Health Rep..

[9] Joubert B.R., Felix J.F., Yousefi P., Bakulski K.M., Just A.C., Breton C., Reese S.E., Markunas C.A., Richmond R.C., Xu C.J. (2016). DNA Methylation in Newborns and Maternal Smoking in Pregnancy: Genome-wide Consortium Meta-analysis. Am. J. Hum. Genet..

[10] Joubert B.R., Haberg S.E., Nilsen R.M., Wang X., Vollset S.E., Murphy S.K., Huang Z., Hoyo C., Midttun O., Cupul-Uicab L.A. (2012). 450K epigenome-wide scan identifies differential DNA methylation in newborns related to maternal smoking during pregnancy. Environ. Health Perspect..

[11] Rogers J.M. (2019). Smoking and pregnancy: Epigenetics and developmental origins of the metabolic syndrome. Birth Defects Res..

[12] Green B.B., Marsit C.J. (2015). Select Prenatal Environmental Exposures and Subsequent Alterations of Gene-Specific and Repetitive Element DNA Methylation in Fetal Tissues. Curr. Environ. Health Rep..

[13] Fragou D., Pakkidi E., Aschner M., Samanidou V., Kovatsi L. (2019). Smoking and DNA methylation: Correlation of methylation with smoking behavior and association with diseases and fetus development following prenatal exposure. Food Chem. Toxicol..

[14] Breton C.V., Byun H.M., Wenten M., Pan F., Yang A., Gilliland F.D. (2009). Prenatal tobacco smoke exposure affects global and gene-specific DNA methylation. Am. J. Respir. Crit. Care Med..

[15] Breton C.V., Siegmund K.D., Joubert B.R., Wang X., Qui W., Carey V., Nystad W., Haberg S.E., Ober C., Nicolae D. (2014). Prenatal tobacco smoke exposure is associated with childhood DNA CpG methylation. PLoS ONE.

[16] Suter M., Ma J., Harris A., Patterson L., Brown K.A., Shope C., Showalter L., Abramovici A., Aagaard-Tillery K.M. (2011). Maternal tobacco use modestly alters correlated epigenome-wide placental DNA methylation and gene expression. Epigenetics.

[17] Suter M., Abramovici A., Showalter L., Hu M., Shope C.D., Varner M., Aagaard-Tillery K. (2010). In utero tobacco exposure epigenetically modifies placental CYP1A1 expression. Metabolism.

[18] Bakulski K.M., Blostein F., London S.J. (2023). Linking Prenatal Environmental Exposures to Lifetime Health with Epigenome-Wide Association Studies: State-of-the-Science Review and Future Recommendations. Environ. Health Perspect..

[19] Sikdar S., Joehanes R., Joubert B.R., Xu C.J., Vives-Usano M., Rezwan F.I., Felix J.F., Ward J.M., Guan W., Richmond R.C. (2019). Comparison of smoking-related DNA methylation between newborns from prenatal exposure and adults from personal smoking. Epigenomics.

[20] Chatterjee A., Rodger E.J., Stockwell P.A., Le Mée G., Morison I.M., Seymour G., Cullinan M., Heng N. (2017). Generating Multiple Base-Resolution DNA Methylomes Using Reduced Representation Bisulfite Sequencing. Oral Biology Molecular Techniques and Applications.

[21] Chatterjee A., Stockwell P.A., Horsfield J.A., Morison I.M., Nakagawa S. (2014). Base-resolution DNA methylation landscape of zebrafish brain and liver. Genom. Data.

[22] Chatterjee A., Rodger E.J., Stockwell P.A., Weeks R.J., Morison I.M. (2012). Technical considerations for reduced representation bisulfite sequencing with multiplexed libraries. J. Biomed. Biotechnol..

[23] Wang J., Xia Y., Li L., Gong D., Yao Y., Luo H., Lu H., Yi N., Wu H., Zhang X. (2013). Double restriction-enzyme digestion improves the coverage and accuracy of genome-wide CpG methylation profiling by reduced representation bisulfite sequencing. BMC Genom..

[24] Campagna M.P., Xavier A., Lechner-Scott J., Maltby V., Scott R.J., Butzkueven H., Jokubaitis V.G., Lea R.A. (2021). Epigenome-wide association studies: Current knowledge, strategies and recommendations. Clin. Epigenetics.

[25] Carmona J.J., Accomando W.P., Binder A.M., Hutchinson J.N., Pantano L., Izzi B., Just A.C., Lin X., Schwartz J., Vokonas P.S. (2017). Empirical comparison of reduced representation bisulfite sequencing and Infinium BeadChip reproducibility and coverage of DNA methylation in humans. npj Genom. Med..

[26] Nakabayashi K., Yamamura M., Haseagawa K., Hata K., Hatada I., Horii T. (2023). Reduced Representation Bisulfite Sequencing (RRBS). Epigenomics Methods in Molecular Biology.

[27] Illingworth R.S., Bird A.P. (2009). CpG islands—‘A rough guide’. FEBS Lett..

[28] Saxonov S., Berg P., Brutlag D.L. (2006). A genome-wide analysis of CpG dinucleotides in the human genome distinguishes two distinct classes of promoters. Proc. Natl. Acad. Sci. USA.

[29] Deaton A.M., Bird A. (2011). CpG islands and the regulation of transcription. Genes Dev..

[30] Illingworth R.S., Gruenewald-Schneider U., Webb S., Kerr A.R., James K.D., Turner D.J., Smith C., Harrison D.J., Andrews R., Bird A.P. (2010). Orphan CpG islands identify numerous conserved promoters in the mammalian genome. PLoS Genet..

[31] Beckett S.M., Laughton S.J., Pozza L.D., McCowage G.B., Marshall G., Cohn R.J., Milne E., Ashton L.J. (2008). Buccal swabs and treated cards: Methodological considerations for molecular epidemiologic studies examining pediatric populations. Am. J. Epidemiol..

[32] Smith A.K., Kilaru V., Klengel T., Mercer K.B., Bradley B., Conneely K.N., Ressler K.J., Binder E.B. (2015). DNA extracted from saliva for methylation studies of psychiatric traits: Evidence tissue specificity and relatedness to brain. Am. J. Med. Genet. B Neuropsychiatr. Genet..

[33] McEwen L.M., O’Donnell K.J., McGill M.G., Edgar R.D., Jones M.J., MacIsaac J.L., Lin D.T.S., Ramadori K., Morin A., Gladish N. (2020). The PedBE clock accurately estimates DNA methylation age in pediatric buccal cells. Proc. Natl. Acad. Sci. USA.

[34] Lowe R., Gemma C., Beyan H., Hawa M.I., Bazeos A., Leslie R.D., Montpetit A., Rakyan V.K., Ramagopalan S.V. (2013). Buccals are likely to be a more informative surrogate tissue than blood for epigenome-wide association studies. Epigenetics.

[35] Rapado-González Ó., Salta S., López-López R., Henrique R., Suárez-Cunqueiro M.M., Jerónimo C. (2024). DNA methylation markers for oral cancer detection in non- and minimally invasive samples: A systematic review. Clin. Epigenetics.

[36] Adeoye J., Alade A.A., Zhu W.Y., Wang W., Choi S.W., Thomson P. (2022). Efficacy of hypermethylated DNA biomarkers in saliva and oral swabs for oral cancer diagnosis: Systematic review and meta-analysis. Oral Dis..

[37] DNA Genotek ORAcollect DxOCD-100/100A. https://www.dnagenotek.com/us/products/collection-human/oracollect-dx/OCD-100.html.

[38] Koni A.C., Scott R.A., Wang G., Bailey M.E., Peplies J., Bammann K., Pitsiladis Y.P., Consortium I. (2011). DNA yield and quality of saliva samples and suitability for large-scale epidemiological studies in children. Int. J. Obes..

[39] Lunatic UV/Vis Spectrophotometer. https://www.unchainedlabs.com/lunatic/.

[40] A Quick Guide for DNA Methylation Profiling with NGS-based Methods. https://www.zymoresearch.com/pages/how-to-detect-dna-methylation.

[41] Akalin A., Kormaksson M., Li S., Garrett-Bakelman F.E., Figueroa M.E., Melnick A., Mason C.E. (2012). methylKit: A comprehensive R package for the analysis of genome-wide DNA methylation profiles. Genome Biol..

[42] Feng H., Wu H. (2019). Differential methylation analysis for bisulfite sequencing using DSS. Quant. Biol..

[43] Feng H., Conneely K.N., Wu H. (2014). A Bayesian hierarchical model to detect differentially methylated loci from single nucleotide resolution sequencing data. Nucleic Acids Res..

[44] Park Y., Wu H. (2016). Differential methylation analysis for BS-seq data under general experimental design. Bioinformatics.

[45] Wu H., Xu T., Feng H., Chen L., Li B., Yao B., Qin Z., Jin P., Conneely K.N. (2015). Detection of differentially methylated regions from whole-genome bisulfite sequencing data without replicates. Nucleic Acids Res..

[46] Wu H., Wang C., Wu Z. (2013). A new shrinkage estimator for dispersion improves differential expression detection in RNA-seq data. Biostatistics.

[47] Genome Browser. https://genome.ucsc.edu/.

[48] Lowdon R.F., Jang H.S., Wang T. (2016). Evolution of Epigenetic Regulation in Vertebrate Genomes. Trends Genet..

[49] GeneCards(R) The Human Gene Database. CALN1 Gene—Calneuron 1. https://www.genecards.org/cgi-bin/carddisp.pl?gene=CALN1.

[50] GeneCards(R) The Human Gene Database. TBL1XR1 Gene—TBL1X/Y Related 1. https://www.genecards.org/cgi-bin/carddisp.pl?gene=TBL1XR1.

[51] OMIM® Online Mendelian Inheritance in Man®. 609791. LEUCINE-RICH REPEAT- AND Ig DOMAIN-CONTAINING NOGO RECEPTOR-INTERACTING PROTEIN 1; LINGO1. https://www.omim.org/entry/609791?search=LINGO&highlight=lingo.

[52] Wu Y.Q., Lin X., Liu C.M., Jamrich M., Shaffer L.G. (2001). Identification of a human brain-specific gene, calneuron 1, a new member of the calmodulin superfamily. Mol. Genet. Metab..

[53] Roderick H.L., Cook S.J. (2008). Ca2+ signalling checkpoints in cancer: Remodelling Ca2+ for cancer cell proliferation and survival. Nat. Rev. Cancer.

[54] Shapovalov G., Ritaine A., Skryma R., Prevarskaya N. (2016). Role of TRP ion channels in cancer and tumorigenesis. Semin. Immunopathol..

[55] Takagi K., Naruse A., Akita K., Muramatsu-Maekawa Y., Kawase K., Koie T., Horie M., Kikuchi A. (2022). CALN1 hypomethylation as a biomarker for high-risk bladder cancer. BMC Urol..

[56] Wang J., Su Y., Tian Y., Ding Y., Wang X. (2019). Characterization of DNA hydroxymethylation profile in cervical cancer. Artif. Cells Nanomed. Biotechnol..

[57] Siedlinski M., Cho M.H., Bakke P., Gulsvik A., Lomas D.A., Anderson W., Kong X., Rennard S.I., Beaty T.H., Hokanson J.E. (2011). Genome-wide association study of smoking behaviours in patients with COPD. Thorax.

[58] OMIM® Online Mendelian Inheritance in Man®. 608628. TRANSDUCIN-BETA-LIKE 1 RECEPTOR 1; TBL1XR1. https://www.omim.org/entry/608628.

[59] Arroyo Carrera I., Fernandez-Burriel M., Lapunzina P., Tenorio J.A., Garcia Navas V.D., Marquez Isidro E. (2021). TBL1XR1 associated intellectual disability, a new missense variant with dysmorphic features plus autism: Expanding the phenotypic spectrum. Clin. Genet..

[60] Zhao Y., Lin H., Jiang J., Ge M., Liang X. (2019). TBL1XR1 as a potential therapeutic target that promotes epithelial-mesenchymal transition in lung squamous cell carcinoma. Exp. Ther. Med..

[61] Heinen C.A., Jongejan A., Watson P.J., Redeker B., Boelen A., Boudzovitch-Surovtseva O., Forzano F., Hordijk R., Kelley R., Olney A.H. (2016). A specific mutation in TBL1XR1 causes Pierpont syndrome. J. Med. Genet..

[62] Nagy A., Molay F., Hargadon S., Brito Pires C., Grant N., De La Rosa Abreu L., Chen J.Y., D’Souza P., Macnamara E., Tifft C. (2024). The spectrum of neurological presentation in individuals affected by TBL1XR1 gene defects. Orphanet J. Rare Dis..

[63] Zhou Z.D., Sathiyamoorthy S., Tan E.K. (2012). LINGO-1 and Neurodegeneration: Pathophysiologic Clues for Essential Tremor. Tremor Other Hyperkinet. Mov..

[64] Llorens F., Gil V., Iraola S., Carim-Todd L., Marti E., Estivill X., Soriano E., del Rio J.A., Sumoy L. (2008). Developmental analysis of Lingo-1/Lern1 protein expression in the mouse brain: Interaction of its intracellular domain with Myt1l. Dev. Neurobiol..

[65] Ansar M., Riazuddin S., Sarwar M.T., Makrythanasis P., Paracha S.A., Iqbal Z., Khan J., Assir M.Z., Hussain M., Razzaq A. (2018). Biallelic variants in LINGO1 are associated with autosomal recessive intellectual disability, microcephaly, speech and motor delay. Genet. Med..

[66] Mahabee-Gittens E.M., Kline-Fath B.M., Harun N., Folger A.T., He L., Parikh N.A. (2023). Prenatal tobacco smoke exposure and risk of brain abnormalities on magnetic resonance imaging at term in infants born very preterm. Am. J. Obstet. Gynecol. MFM.

[67] Mahabee-Gittens E.M., Harun N., Glover M., Folger A.T., Parikh N.A. (2024). Cincinnati Infant Neurodevelopment Early Prediction Study Investigators. Prenatal tobacco smoke exposure and risk for cognitive delays in infants born very premature. Sci. Rep..

[68] Wang L., Sun J., Wu H., Liu S., Wang J., Wu B., Huang S., Li N., Wang J., Zhang X. (2012). Systematic assessment of reduced representation bisulfite sequencing to human blood samples: A promising method for large-sample-scale epigenomic studies. J. Biotechnol..

[69] Attree E., Griffiths B., Panchal K., Xia D., Werling D., Banos G., Oikonomou G., Psifidi A. (2024). Identification of DNA methylation markers for age and Bovine Respiratory Disease in dairy cattle: A pilot study based on Reduced Representation Bisulfite Sequencing. Commun. Biol..

[70] Karami K., Sabban J., Cerutti C., Devailly G., Foissac S., Gourichon D., Hubert A., Hubert J.N., Leroux S., Zerjal T. (2025). Molecular responses of chicken embryos to maternal heat stress through DNA methylation and gene expression: A pilot study. Environ. Epigenet..

[71] Benowitz N.L., Bernert J.T., Foulds J., Hecht S.S., Jacob P., Jarvis M.J., Joseph A., Oncken C., Piper M.E. (2020). Biochemical Verification of Tobacco Use and Abstinence: 2019 Update. Nicotine Tob. Res..

[72] Peacock J.L., Palys T.J., Halchenko Y., Sayarath V., Takigawa C.A., Murphy S.E., Peterson L.A., Baker E.R., Karagas M.R. (2022). Assessing tobacco smoke exposure in pregnancy from self-report, urinary cotinine and NNAL: A validation study using the New Hampshire Birth Cohort Study. BMJ Open.

[73] Smith M.R., Saberi S., Ajaykumar A., Zhu M.M.T., Gadawski I., Sattha B., Maan E.J., Van Shalkwyk J., Elwood C., Pick N. (2023). Robust tobacco smoking self-report in two cohorts: Pregnant women or men and women living with or without HIV. Sci. Rep..

[74] Arbuckle T.E., Liang C.L., Fisher M., Caron N.J., Fraser W.D., MIREC Study Group (2018). Exposure to tobacco smoke and validation of smoking status during pregnancy in the MIREC study. J. Expo. Sci. Environ. Epidemiol..

